# Preoperative TAPSE/PASP Ratio as a Non-Invasive Predictor of Hypotension After General Anesthesia Induction

**DOI:** 10.3390/diagnostics15111404

**Published:** 2025-05-31

**Authors:** Ferdi Gülaştı, Sevil Gülaştı, Büşra Ceyhan Can, Hakan Öztürk, Sinem Sarı

**Affiliations:** 1 Department of Anesthesiology and Reanimation, Faculty of Medicine, Adnan Menderes University, Aydın 09010, Türkiye; drbusracan@hotmail.com (B.C.C.); sarisinem@yahoo.com (S.S.); 2 Department of Cardiology, Faculty of Medicine, Adnan Menderes University, Aydın 09010, Türkiye; drsevilonay@hotmail.com; 3 Department of Biostatistics, Faculty of Medicine, Adnan Menderes University, Aydın 09010, Türkiye; h.ozturk3509@gmail.com; 4 Outcomes Research Consortium, Houston, TX 77030, USA

**Keywords:** TAPSE/PASP ratio, general anesthesia, right ventricle, echocardiography, hypotension

## Abstract

**Background:** Hypotension is a common adverse event after the induction of general anesthesia and may lead to serious complications. The Tricuspid Annular Plane Systolic Excursion (TAPSE)/Pulmonary Arterial Systolic Pressure (PASP) ratio is an echocardiographic parameter reflecting right ventricular (RV) function and pulmonary circulation. This study aimed to evaluate the predictive value of the TAPSE/PASP ratio for hypotension after general anesthesia induction. **Methods:** This prospective observational study included 79 patients with no known cardiac disease who were scheduled for elective surgery and classified as having a physical status of I–III according to the American Society of Anesthesiologists (ASA). TAPSE, PASP, and RV function were assessed using transthoracic echocardiography (TTE) within 5–30 min before surgery, and their hemodynamic changes after general anesthesia induction were recorded. **Results:** Data analysis revealed a significant association between the TAPSE/PASP ratio and the occurrence of hypotension following the induction of general anesthesia (*p* < 0.001). In addition, a cut-off value of ≤1.98 was determined for predicting hypotension, which demonstrated a sensitivity of 72.5% and a specificity of 64.1% (AUC = 0.733, 95% CI: 0.621–0.826, *p* < 0.001). **Conclusions:** The TAPSE/PASP ratio is a potential predictor of hypotension following the induction of general anesthesia. Further studies are required to validate its predictive accuracy and clinical utility in perioperative hemodynamic management.

## 1. Introduction

Intraoperative hypotension is a major contributor to morbidity and mortality, as it can lead to organ damage, including myocardial injury, acute kidney injury, and stroke, as well as prolonged hospital stays [1,2,3,4,5,6,7]. In this regard, general anesthesia, an essential component of major surgeries, has been found to cause hypotension due to the cardiovascular depressant effects of anesthetic agents and the reduction in preload caused by positive-pressure ventilation [8,9,10,11,12]. Thus, identifying patients at risk of post-induction hypotension is crucial for facilitating early intervention and optimizing perioperative management. Various hemodynamic parameters, such as inferior vena cava (IVC) diameter, IVC collapsibility index (CI), and internal jugular vein area, have been investigated as potential predictors. However, related practical and cost-effective methods for risk stratification remain investigational or lack robust validation [13,14].

Tricuspid Annular Plane Systolic Excursion (TAPSE) is a widely recognized echocardiographic parameter used for the evaluation of right ventricular (RV) systolic function. It reflects the longitudinal movement of the tricuspid annulus toward the apex of the heart during systole, which serves as an indirect marker of RV contractility [15]. Given the complex geometry of the right ventricle, which poses significant challenges for accurate volumetric assessment, Tricuspid Annular Plane Systolic Excursion provides a practical alternative that is both reproducible and clinically meaningful. One of the major advantages of Tricuspid Annular Plane Systolic Excursion is its relative independence from overall image quality and reduced susceptibility to inter- and intra-observer variability [15]. It is measured in the apical four-chamber view using M-mode echocardiography, and its acquisition is straightforward, rapid, and feasible even in critically ill patients or in those with suboptimal imaging conditions. A Tricuspid Annular Plane Systolic Excursion value of less than 17 mm is considered to be indicative of impaired right ventricular systolic function and has been associated with adverse clinical outcomes in a variety of cardiovascular conditions, including pulmonary hypertension, heart failure, and pulmonary embolism [15]. Due to its simplicity, non-invasiveness, and prognostic relevance, Tricuspid Annular Plane Systolic Excursion has become an integral component of routine echocardiographic assessment in contemporary cardiology practice.

Pulmonary artery pressure is determined by the amount of blood flowing in the pulmonary circulation and the intrinsic properties of the cardiovascular system [16]. Due to both pulmonary pathogens and left ventricular dysfunction, pulmonary circulation resistance is possible. Pulmonary artery systolic pressure can be calculated by Doppler echocardiography from the permanent systolic right ventricular to right atrial pressure gradient using the modified Bernoulli equation (4 × peak tricuspid regurgitation velocity squared). Pulmonary artery systolic pressure is studied echocardiographically by adding the estimated right atrial pressure [16].

Recent studies have demonstrated that the Tricuspid Annular Plane Systolic Excursion (TAPSE)/Pulmonary Arterial Systolic Pressure (PASP) ratio, which reflects right ventricular (RV) to pulmonary artery (PA) coupling, is associated with the prognosis of pulmonary hypertension (PHT) and heart failure (HF) [17,18]. As RV–PA coupling quantitatively represents the volume adaptation of the RV, it provides valuable insight into right heart function. Given that many studies investigating the prediction of hypotension following anesthesia induction have focused on assessing patients’ volumetric status, the TAPSE/PASP ratio may serve as a potential indicator of hemodynamic stability. Based on this rationale, the present study evaluated the utility of the TAPSE/PASP ratio in predicting hypotension following general anesthesia induction, considering its ability to reflect RV volume adaptation.

## 2. Materials and Methods

### 2.1. Study Design and Ethical Approval

This prospective observational study was conducted between June 2024 and January 2025, following approval from the Adnan Menderes University Faculty of Medicine Non-Invasive Clinical Research Ethics Committee (Protocol No: 2024-88; 5 May 2024; Aydın, Türkiye). The study was registered in the ClinicalTrials.gov database on 10 May 2024 (NCT06414811). Written informed consent was obtained from all participants. A data analysis plan was posted on a publicly accessible server (ClinicalTrials.gov), and a statistical analysis plan was prepared and securely filed.

### 2.2. Study Participants

A total of 79 patients, aged between 18 and 75, who were scheduled for elective surgery under general anesthesia were included in this study. In the selection of participants, individuals between ASA I and ASA III according to the physical status classification of the American Society of Anesthesiologists (ASA) were found suitable. In addition, the criterion of not having any known cardiac disease was taken as the basis for the patients being included in the study. In this way, we attempted to minimize the potential effect of underlying heart diseases that could significantly affect cardiac function in the results.

Accordingly, pregnant individuals, patients who had previously undergone cardiac surgery, individuals diagnosed with severe pulmonary hypertension (PHT), those with advanced valve disease, those with structural heart diseases such as hypertrophic or dilated cardiomyopathy, patients who were experiencing or had recently experienced acute myocardial infarction, and individuals with a rhythm other than sinus rhythm were excluded from the study.

### 2.3. Transthoracic Echocardiography (TTE) and Data Collection

Transthoracic echocardiography (TTE) was performed within 30 min before anesthesia induction, while the patients were completely fasting. All measurements were recorded during this period. Demographic characteristics, preoperative laboratory data, and cardiac parameters obtained with TTE were systematically collected for each patient. In addition, basic hemodynamic parameters were recorded every two minutes from anesthesia induction to surgical incision in order to monitor hemodynamic changes. The patients were kept in a supine position throughout the study. In this way, the possible effects of postural changes on hemodynamic data were minimized. Only minimal external stimuli were allowed during the postanesthesia period. These stimuli included low-intensity stimuli such as the placement of a urinary catheter and the preparation of the surgical field with antiseptic solutions.

### 2.4. Definition of Hypotension and Hemodynamic Management

Hypotension was defined as a decrease of 30% or more in systolic blood pressure (SBP) compared to baseline, a decrease in SBP below 90 mmHg, or a decrease in mean arterial pressure (MBP) below 60 mmHg. In cases where the ambient pressure fell below 55 mmHg or remained below 60 mmHg for more than two minutes, hypotension was treated with intravenous ephedrine (0.1 mg/kg). According to these criteria, patients were divided into two groups, as those who developed hypotension and those who did not. In line with the main objective of this study, the performance of the TAPSE/PASP ratio, calculated using tricuspid annular planysystolic excursion (TAPSE) and pulmonary artery systolic pressure (PASP) values obtained by transthoracic echocardiography, in predicting hypotension following anesthesia induction was evaluated.

### 2.5. Anesthesia Induction and Monitoring

All patients underwent continuous monitoring, including electrocardiography (ECG), pulse oximetry, and capnography, from the initiation of manual ventilation. Non-invasive blood pressure monitoring was performed using oscillometry, with additional invasive arterial blood pressure monitoring when required by the surgical protocol. In addition, non-invasive blood pressure measurements were taken at two-minute intervals until the start of surgery. Before induction, the patients received normal saline infusion at a rate of 10 mL/kg/hour. The first post-induction blood pressure measurement was recorded before tracheal intubation. Anesthesia was induced using fentanyl (1 μg/kg), lidocaine (1 mg/kg), propofol (2 mg/kg), and rocuronium (0.6 mg/kg). After intubation, anesthesia was maintained with sevoflurane at 2%.

When spontaneous respiration was lost after anesthesia induction, patients were ventilated via mask, followed by volume-controlled positive-pressure ventilation (PPV) initiated after endotracheal intubation with a standard PEEP of 5 cm H_2_O and a tidal volume target of 6–8 mL/kg. The inspiratory oxygen fraction (FiO_2_) was adjusted to maintain SpO_2_ between 95% and 99%.

### 2.6. Echocardiography

All patients underwent TTE in the left lateral decubitus position using a GE Vivid E9 echocardiography system. A single trained observer performed all measurements, ensuring consistency. The mean values of three cardiac cycles were recorded for each parameter. Measurement techniques adhered to the guidelines of the American Society of Echocardiography. Standard echocardiographic assessments included M-mode, two-dimensional, color Doppler, pulse-wave Doppler, continuous-wave Doppler, and tissue Doppler imaging (TDI). The left ventricular (LV) ejection fraction (EF) was evaluated in the parasternal long-axis view.

**Figure 1:**
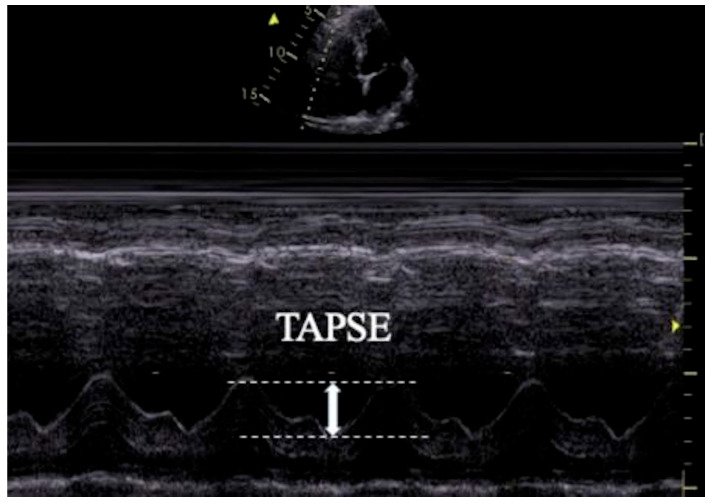
Transthoracic echocardiographic image illustrating TAPSE measurement.

TAPSE was measured in the apical four-chamber view by placing an M-mode cursor across the tricuspid annulus and recording the peak systolic longitudinal displacement. The average value of five consecutive cardiac cycles was documented (Figure 1).

**Figure 2:**
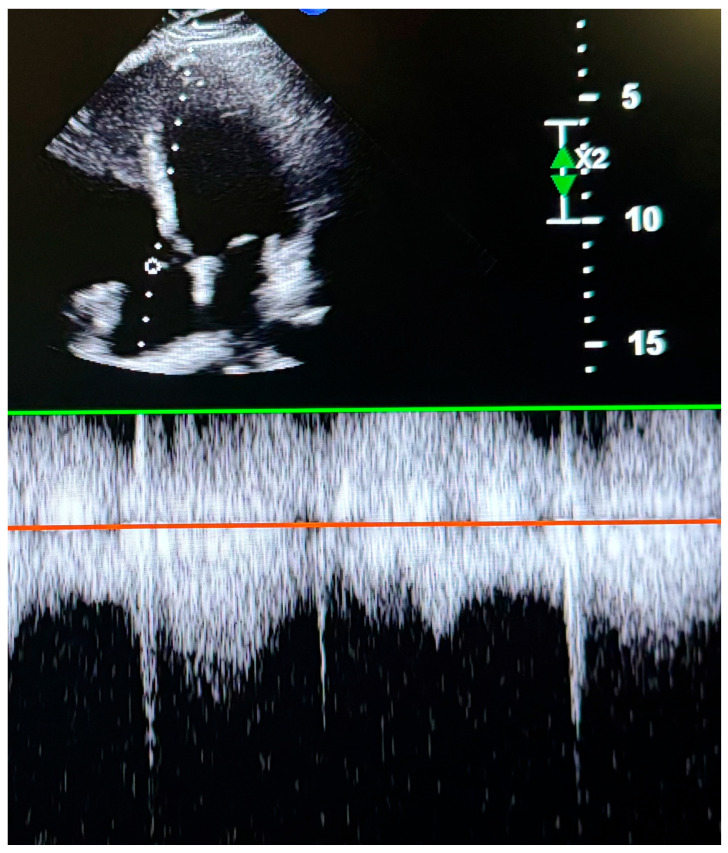
Transthoracic echocardiographic image showing tricuspid peak velocity.

**Figure 3:**
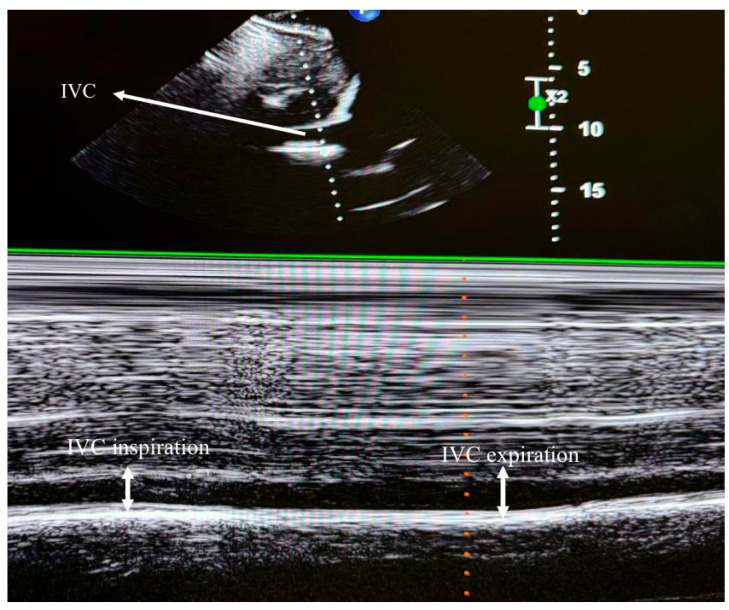
Transthoracic echocardiographic image depicting inferior vena cava (IVC) diameters.

To assess RV systolic function, myocardial performance index (MPI), fractional area change (FAC), TAPSE, and TDI-derived indices were measured. RV diastolic function was evaluated using tricuspid E and A wave velocities and the E/A ratio. TAPSE was obtained by placing an M-mode marker along the tricuspid annulus and measuring its longitudinal motion at peak systole. Ejection time (ET), isovolumetric relaxation time (IVRT), and isovolumetric contraction time (IVCT) were derived from TDI images. The MPI was calculated as a global index of RV function using the formula MPI = (IVCT + IVRT)/ET. Tricuspid regurgitation (TR) was assessed using color Doppler interrogation in the apical four-chamber view. A well-defined continuous-wave (CW) Doppler TR signal was obtained for the accurate determination of the peak tricuspid regurgitant velocity (TRV) (Figure 2). IVC measurements were performed in the supine position using a subxiphoid approach. The cursor was placed 1 cm distal to the hepatic vein–IVC junction, and the IVC diameter was monitored for 30 s in M-mode. Measurements were recorded at peak inspiration (IVC_ins_) and peak expiration (IVC_exp_) during normal breathing (Figure 3). The IVC collapsibility index (IVC-CI) was calculated using the formula: IVC-CI = (IVC_exp_ − IVC_ins_)/IVC_exp_.

## 3. Sample Size Calculation

Since no previous studies with a similar methodology were available, a post hoc power analysis was performed to evaluate the statistical power achieved in this study. The analysis was conducted using G*Power software (version 3.1.2), based on the observed means and standard deviations of TAPSE/PASP in the groups with and without hypotension. An independent-samples *t*-test (two-tailed) was used, and the effect size (Cohen’s d) calculated from the actual data was 0.88. With an alpha level of 0.05 and group sizes of 39 and 40, the achieved statistical power (1–β) was 0.972, indicating a high probability of detecting the observed effect.

### Statistical Analysis

Statistical analyses were performed using the IBM SPSS Statistics 26 and MedCalc Statistical Software. The Kolmogorov–Smirnov test was used to assess whether the numerical variables followed a normal distribution, which determined the choice between parametric (e.g., independent-samples *t*-test) and non-parametric tests (e.g., Mann–Whitney U test) during comparative analyses. Normally distributed variables are expressed as the mean ± standard deviation (SD), and non-normally distributed variables are presented as the median (25th–75th percentile). Categorical variables are shown as the frequency and percentage. For comparisons between two independent groups, the independent-samples *t*-test was used for normally distributed variables, whereas the Mann–Whitney U test was applied for non-normally distributed variables. The relationships between categorical variables were analyzed using the chi-square test of independence. Receiver operating characteristic (ROC) analysis was conducted using the DeLong method with the Youden index to determine the optimal cut-off value of the TAPSE/PASP ratio for predicting hypotension. A *p*-value < 0.05 was considered statistically significant.

## 4. Results

**Chart 1:**
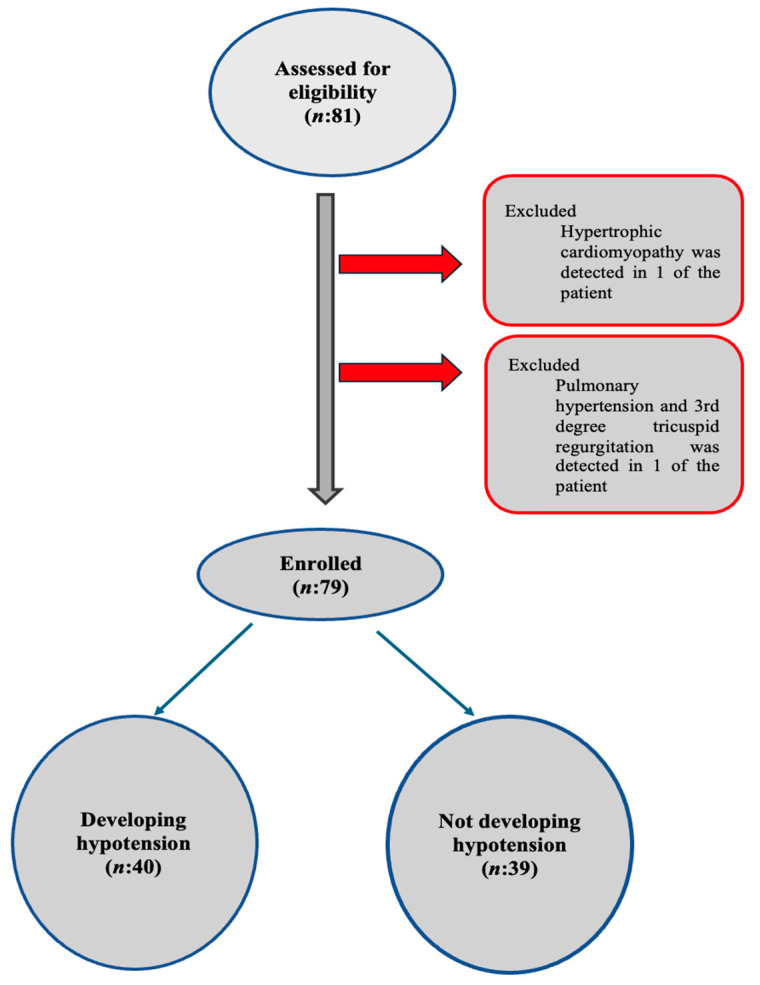
Flow diagram.

A total of 81 patients were enrolled in the study. One patient was excluded due to hypertrophic cardiomyopathy, and another was excluded after TTE revealed a pulmonary arterial systolic pressure (PASP) of 51 mmHg and grade 3 tricuspid insufficiency (Chart 1).

**Figure 4:**
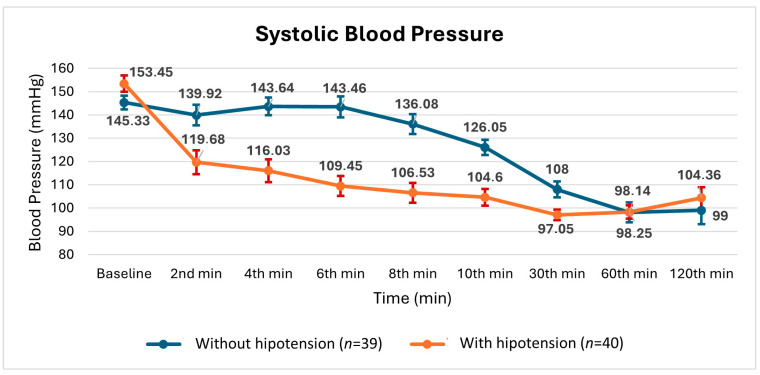
Changes in mean ± SE (standard error) systolic blood pressure measurements during surgery in patients with and without hypotension.

**Figure 5:**
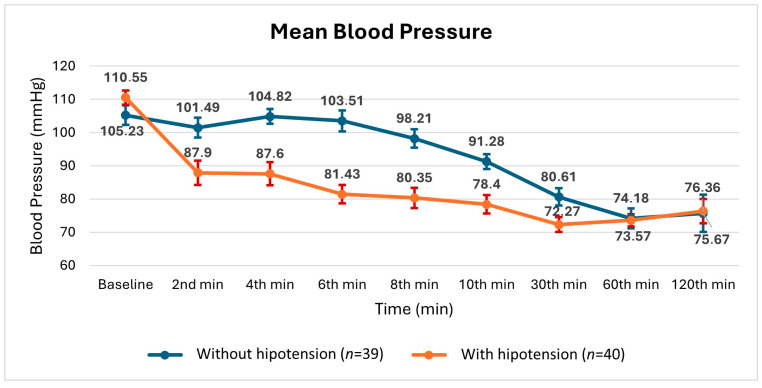
Changes in mean ± SE (standard error) blood pressure measurements during surgery in patients with and without hypotension.

No significant differences were observed between the groups in terms of age, sex distribution, comorbidities, ASA scores, or type of surgical intervention (*p* > 0.05) (Table 1). Similarly, there were no significant differences in laboratory parameters between patients with and without hypotension (*p* > 0.05) (Table 2). The temporal changes in SBP and DBP are illustrated in Figure 4 and Figure 5.

The preoperative TTE parameters are summarized in Table 3. The TAPSE, PASP, and TAPSE/PASP ratio values were significantly lower in patients who developed hypotension compared to those who did not (*p* < 0.001) (Table 3). However, no significant differences were observed between the two groups in terms of LVEF, S’, Tricuspid E, Tricuspid A, TRIC CM SEC, RVEDA, RVEDV, RVESA, and RVESV values (*p* < 0.05) (Table 3).

**Figure 6:**
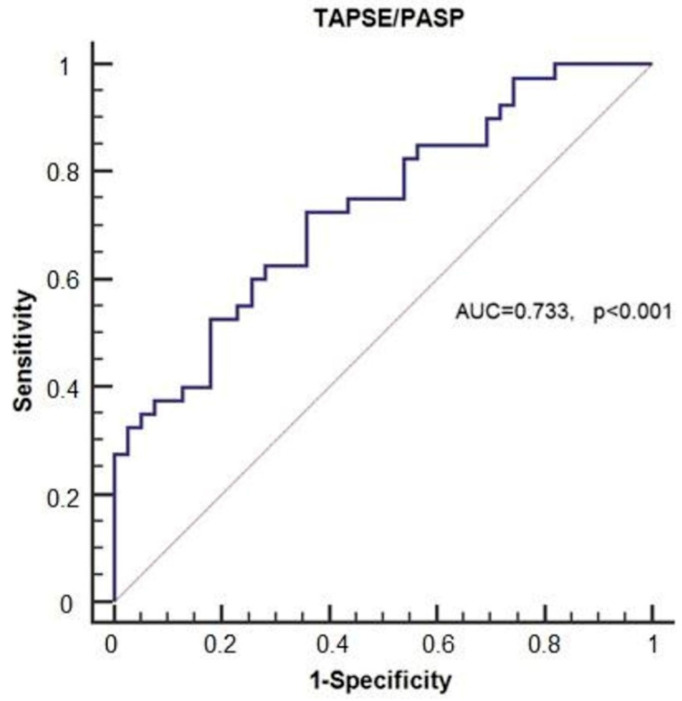
Receiver operating characteristic (ROC) curve illustrating the predictive value of the TAPSE/PASP ratio for hypotension.

The optimal cut-off value of the TAPSE/PASP ratio for predicting hypotension following general anesthesia induction was determined to be ≤1.98 (Figure 6), which demonstrated a sensitivity of 72.5% and a specificity of 64.1%, with an AUC of 0.733 (95% CI: 0.621–0.826, *p* < 0.001) (Table 4).

The optimal cut-off value of the TAPSE/PASP ratio for predicting hypotension following general anesthesia induction was determined to be ≤1.98 using ROC curve analysis and the Youden index, which identifies the point maximizing the combined sensitivity and specificity. At this threshold, the TAPSE/PASP ratio demonstrated a sensitivity of 72.5% and a specificity of 64.1% for identifying patients who developed hypotension (Figure 6), with an area under the curve (AUC) of 0.733 (95% CI: 0.621–0.826, *p* < 0.001) (Table 4). In this context, sensitivity refers to the proportion of hypotensive patients correctly identified by the cut-off, while specificity reflects the proportion of non-hypotensive patients correctly excluded.

The optimal cut-off value of TAPSE for predicting hypotension following general anesthesia induction was determined to be ≤23.13, and this threshold demonstrated a sensitivity of 72.5% and a specificity of 79.5%, with an AUC of 0.807 (95% CI: 0.703–0.887, *p* < 0.001) (Table 4).

## 5. Discussion

This prospective observational study evaluated the predictive value of the preoperative TAPSE/PASP ratio in distinguishing patients at risk of hypotension after the induction of general anesthesia, and the findings demonstrated that a TAPSE/PASP ratio of ≤1.98 predicted post-induction hypotension with a sensitivity of 72.5% and a specificity of 64.1%.

Under normal physiological conditions, the pulmonary circulation presents a low-resistance environment, allowing the RV to pump blood into the lungs with minimal effort. To maintain efficient blood flow, the contractile strength of the RV is naturally aligned with the properties of the pulmonary arteries, a relationship known as RV–PA coupling. RV–PA coupling can be invasively derived through right heart catheterization by calculating the ratio between ventricular elastance and arterial elastance.

In a study conducted by Tello et al., a strong correlation was demonstrated between RV–PA coupling, traditionally assessed invasively via right heart catheterization, and the non-invasive TAPSE/PASP ratio, measured by echocardiography [19]. Similarly, in a study conducted by Hui Li et al. [20], TAPSE was found to be significantly higher in patients with an atrial septal defect (ASD) who exhibited an increased right ventricular (RV) volume, as well as in the pulmonary hypertension (PHT) and control groups. These findings indicate that TAPSE is influenced not only by RV systolic function but also by RV volume loading. Furthermore, the same study reported that the TAPSE/PASP ratio was significantly lower in both ASD and PHT patient groups when compared to healthy controls, suggesting that this parameter may be a more comprehensive marker reflecting both RV function and afterload [20].

RV–PA coupling has recently been recognized as an important prognostic marker in HF and PHT [21,22,23]. The gold standard for assessing RV–PA coupling is the ratio of right ventricular end-systolic elastance to effective arterial elastance (Ees/Ea), the measurement of which requires invasive measurements and specialized equipment, limiting its routine clinical use [24]. As a non-invasive alternative, the TAPSE/PASP ratio has been proposed to evaluate RV–PA coupling [19,20,21]. It integrates TAPSE, a marker of RV longitudinal shortening, with PASP, an indicator of afterload, providing a comprehensive assessment of RV contractility and load adaptation beyond the isolated interpretation of either parameter [19,21]. In addition to RV function, preoperative volume status and the vasodilatory effects of anesthesia agents play a crucial role in the development of hypotension after anesthesia induction. Given this hemodynamic interplay, the present study investigated the predictive value of the TAPSE/PASP ratio for post-induction hypotension, demonstrating a significant association. A TAPSE/PASP ratio of ≤1.98 effectively predicted the occurrence of hypotension with a sensitivity of 72.5% and a specificity of 64.1%, highlighting its potential clinical utility in perioperative risk stratification.

Minor variations in the definitions of hypotension have been observed, with thresholds ranging from a mean arterial pressure (MAP) of 60 mmHg to 65 mmHg or an SBP of 90 mmHg to 100 mmHg. In the present study, hypotension was defined as MAP ≤ 65 mmHg, SBP ≤ 90 mmHg, or a ≥30% reduction in baseline SBP.

As shown in Figure 4 and Figure 5, the systolic and mean blood pressures at 30 min were found to be similar between the groups with and without hypotension. Considering the preparations for the surgical procedures listed in Table 1, the effect of preoperative hypovolemia was considered limited.

General anesthesia induction has a depressive effect on right ventricular (RV) function. During induction, increased pulmonary vascular resistance associated with atelectasis, hypercapnia, or hypoxia may impose additional loading on the right ventricle. During this period, both mask ventilation and positive-pressure ventilation after endotracheal intubation affect right ventricular filling pressures. Combined with PPV, propofol causes a decrease in cardiac preload through the dilation of venous capacitance vessels and a reduction in sympathetic activity, leading to hypotension [25,26,27]. Remifentanil, at moderate doses, does not affect cardiac filling pressures, stroke volume, or cardiac output but may reduce heart rate and systemic vascular resistance [28,29]. It is well known that tracheal intubation can trigger autonomic responses leading to hypertension or tachycardia. In our study, to control these hemodynamic reactions, premedication with remifentanil and lidocaine was administered. Additionally, the depth of anesthesia was carefully adjusted during intubation, and rapid sequence intubation techniques were applied to minimize autonomic responses. These measures helped to limit blood pressure fluctuations related to intubation.

Several studies have investigated the use of IVC ultrasound measurements to predict post-induction hypotension. A meta-analysis by Ella Dana et al., including 14 studies, reported sensitivity and specificity values ranging from 45.5% to 86.67% and 77.27% to 94.29%, respectively [30]. In contrast, Omar H et al. found no significant differences between patients with and without hypotension when using IVC-CI measurements to predict post-induction hypotension [27]. Consistent with these findings, the present study did not identify significant differences in IVC-CI values between the hypotensive and non-hypotensive groups. In our study, TAPSE at a cut-off value of ≤23.13 cm demonstrated a sensitivity of 72.5% and specificity of 79.5%. In our study, TAPSE/PASP at a cut-off value of 1.98 cm demonstrated a sensitivity of 72.5% and specificity of 64.1%. When IVC-CI and TAPSE/PASP are compared with each other, the sensitivity and specificity of TAPSE/PASP seem to be better.

The present study had several limitations. First, blood pressure was measured non-invasively at two-minute intervals, which may have limited the precision of hypotension detection. Second, the majority of participants had an ASA physical status of I–II, potentially restricting the generalizability of these findings to higher-risk populations. Despite these limitations, this study provides new insights into the predictive value of the TAPSE/PASP ratio for post-induction hypotension. Given that this is the first study to evaluate the TAPSE/PASP ratio for this purpose, further research is warranted to validate these findings and explore their clinical applications.

## 6. Conclusions

The findings of this study indicate that a TAPSE/PASP ratio of ≤1.98, measured preoperatively via TTE, serves as an independent predictor of hypotension following the induction of general anesthesia. Given its non-invasive nature and potential clinical utility, the TAPSE/PASP ratio may aid in perioperative risk assessment and hemodynamic optimization. However, further studies are required to validate its diagnostic accuracy and establish its role in clinical practice.

### Key Messages

- RV–PA coupling quantitatively reflects right ventricular volume adaptation.
- The TAPSE/PASP ratio is a predictive marker for hypotension following general anesthesia induction.
- A TAPSE/PASP ratio of ≤1.98 demonstrated a sensitivity of 72.5% and a specificity of 64.1%, with an AUC of 0.733 (95% CI: 0.621–0.826, *p* < 0.001).
- A TAPSE of ≤1.98 demonstrated a sensitivity of 72.5% and a specificity of 79.5%, with an AUC of 0.807 (95% CI: 0.703–0.887, *p* < 0.001).
- No significant differences were observed in IVC-CI values between patients with and without hypotension.

## Figures and Tables

**Table 1:** Demographic and clinical characteristics of the patients with and without hypotension.

Variables	Without Hypotension (*n* = 39)	with Hypotension (*n* = 40)	*p*
Age	58.0 (47.0–67.0)	65.0 (53.3–69.0)	0.101
Sex			
Male	26 (66.7)	24 (60.0)	0.703
Female	13 (33.3)	16 (40.0)	
Height	168.97 ± 8.37	164.90 ± 8.99	0.040
Weight	76.13 ± 14.78	72.03 ± 12.16	0.181
BMI	26.52 ± 3.88	26.47 ± 3.85	0.953
Comorbidity			
**DM**			
No	34 (87.2)	34 (85.0)	1.000
Yes	5 (12.8)	6 (15.0)	
**HT**			
No	30 (76.9)	33 (82.5)	0.736
Yes	9 (23.1)	7 (17.5)	
**Hypothyroidisim**			
No	37 (94.9)	38 (95.0)	1.000
Yes	2 (5.1)	2 (5.0)	
**ASA**			
1	24 (61.5)	19 (47.5)	0.305
2	15 (38.5)	21 (52.5)	
**POEM**	2 (5.1)	0 (0)	0.241
**Ophthalmic Surgeon**	34 (87.2)	39 (97.5)	0.108
**CDH surgeon**	3 (7.7)	1 (2.5)	0.359

DM: Diabetes Mellitus, HT: Hypertension, ASA: American Society of Anesthesiologists, POEM: Peroral Endoscopic Myotomy, CDH: Cervical Disc Herniation.

**Table 2:** Comparison of laboratory parameters between patients with and without hypotension.

Variables	Without Hypotension (*n* = 39)	with Hypotension (*n* = 40)	*p*
Leukocyte (mcl)	7.59 (6.40–8.60)	7.31 (6.50–8.39)	0.663
Hemoglobin (g/dL)	13.50 (12.80–15.30)	13.20 (12.30–13.83)	0.089
Urea (mg/dL)	25.00 (21.00–36.00)	27.50 (22.25–39.75)	0.312
Creatinine (mg/dL)	0.88 ± 0.19	0.84 ± 0.25	0.448

**Table 3:** Echocardiographic parameters of patients with and without hypotension.

Variables	Without Hypotension (*n* = 39)	with Hypotension (*n* = 40)	*p*
TAPSE	25.75 ± 3.23	22.17 ± 2.68	<0.001
PASP	11.31 (9.25–14.35)	13.76 (10.56–21.59)	0.026
TAPSE/PASP	2.29 ± 0.81	1.63 ± 0.68	<0.001
LV EF%	65.00 (65.00–68.00)	65.00 (65.00–68.00)	0.186
S’	19.80 ± 4.18	19.98 ± 5.82	0.874
IVRT	71.31 ± 17.73	74.43 ± 17.20	0.430
IVCT	69.00 (60.00–78.00)	63.50 (57.00–78.00)	0.262
ET	208.54 ± 39.86	204.95 ± 34.69	0.670
MPI	0.64 (0.55–0.80)	0.66 (0.58–0.84)	0.576
TAPS Velocity	0.93 (0.72–1.26)	1.09 (0.66–1.83)	0.243
Tricuspid E	52.65 ± 10.09	52.86 ± 12.24	0.934
Tricuspid A	38.00 (33.00–46.00)	41.00 (34.25–48.00)	0.307
Tricuspid E/A	1.32 ± 0.21	1.25 ± 0.28	0.189
RV EDA	25.00 ± 8.63	22.53 ± 7.94	0.189
RV EDV	50.00 (39.00–74.00)	43.45 (33.75–61.56)	0.161
RV ESA	13.09 (10.00–16.76)	12.52 (10.01–15.71)	0.806
FAC	0.44 (0.32–0.55)	0.40 (0.28–0.55)	0.375
IVC inspirium	10.47 ± 3.62	9.73 ± 4.32	0.412
IVC expirium	15.39 ± 3.85	15.46 ± 4.37	0.941
IVC-CI	0.37 (0.22–0.45)	0.38 (0.29–0.51)	0.305

TAPSE: Tricuspid Annular Plane Systolic Excursion, PASP: Pulmonary arterial systolic pressure, LV: Left Ventricular, EF: Ejection Fraction, IVRT: Isovolumetric Relaxation Time, IVCT: Isovolumetric Contraction Time, ET: Ejection Time, MPI: Myocardial Performance Index, RV: Right Ventricular, EDA: End Diastolic Area, EDV: End Diastolic Volume, ESA: End Systolic Area, FAC: Fractional Area Change, IVC: Inferior Vena Cava, CI: Collabsility Index.

**Table 4:** ROC curve analysis of the TAPSE/PASP ratio for predicting hypotension.

Variable	Cut-Off	Sensitivity	Specificity	Youden Index J	AUC (95% CI)	*p*
TAPSE/PASP	≤1.988	0.725	0.641	0.366	0.733 (0.621–0.826)	<0.001
TAPSE	≤23.13	0.725	0.795	0.520	0.807 (0.703–0.887)	<0.001

TAPSE: Tricuspid Annular Plane Systolic Excursion, PASP: Pulmonary arterial systolic pressure.

## Data Availability

The raw data supporting the conclusions of this article will be made available by the authors on request.
